# Identifying environmental and economic development factors in sustainable entrepreneurship over time by partial least squares (PLS)

**DOI:** 10.1371/journal.pone.0238462

**Published:** 2020-09-04

**Authors:** Ismael Moya-Clemente, Gabriela Ribes-Giner, Odette Pantoja-Díaz

**Affiliations:** 1 Faculty of Business Administration and Management, Universitat Politècnica de València, Valencia, Spain; 2 Sistemas de Información, Gestión de la Tecnología e Innovación (SIGTI‐Research Group), Escuela Politécnica Nacional, Quito, Ecuador; Institute for Advanced Sustainability Studies, GERMANY

## Abstract

This study analyses the impact of environmental and economic factors consolidation on sustainable entrepreneurship over time. A model is proposed that analyses the relations between these factors and sustainable entrepreneurship over time with sustainable development goals performances and the continuation of the businesses index as variables. Using data from 50 countries, a quantitative method based on partial least squares was applied to validate the proposed model. Our findings showed positive and significant relations between environmental and economic factors with sustainable entrepreneurship over time. This implies that the countries which invest more efforts to consolidate their economic and environmental factors obtain higher durability rates for their sustainable entrepreneurship.

## 1. Introduction

Nowadays, problems related to environmental, social and economic issues arise worldwide, including issues like global warming, hunger and unemployment [1]. As an alternative to such problems, sustainable development arises, which focuses on searching for alternatives that satisfy current existing needs without compromising the ability of future generations to satisfy their own needs [2].

Along with all this, it has been proven that entrepreneurships constitute effective mechanisms for generating wealth, and are considered drivers of change, innovation and economic growth [3,4]. However, when faced with the relevance of sustainable development and the importance of its adequate implementation, entrepreneurship is seen to have objectives that go beyond economic benefits [5,6]. Moreover, the need for the business models adopted by entrepreneurship to correspond to sustainability has been highlighted to respond to sustainable development goals (SDG) [7]. Thus sustainable entrepreneurship has become relevant, and focuses on preserving nature, supporting life and the community, generating services and products that are reflected in individuals’ well-being, economy and society [5]. Hence sustainable entrepreneurship remains to be seen as a possible solution that contributes to sustainable development [8,9].

It is also worth stressing the importance of the durability and permanence with time that sustainable entrepreneurship must have. One sustainability basis indicates that enviro-economic resources will not be compromised in the long term, where the durability and extension of sustainable practices will become relevant over time. This is why long-term duration is a key element for sustainable entrepreneurship [10]. Research like [1] centres on the business sustainability that an entrepreneur creates if it is to actually last over time. This long-term vision is maintained by [11], who state that sustainable entrepreneurship focuses on preserving nature, life support and the community.

In previous studies, authors have identified combinations of environmental factors and economic development factors that can help increase sustainable entrepreneurship over time [12]. Our key finding was that the sustainable use of terrestrial ecosystems at high levels, sustainable growth, decent work, and good access to affordable sustainable energy and clean water all help to promote sustainable entrepreneurship over time.

Despite the fact that sustainable entrepreneurship is a topic of current interest [5,7], on which numerous research works have been conducted, it research that addresses the durability of such sustainable entrepreneurship is lacking. There are very few works in the literature about the main triggers or conditions that promote lasting sustainable entrepreneurship over time. This is why the objective of our work was to analyse those factors that allow sustainable entrepreneurship to perpetuate over time and to, thus, shed light on this topic. For this reason, the impact of consolidations of economic and environmental factors on sustainable entrepreneurship durability was studied.

Consolidations of economic and environmental factors were analysed through SDG achievements. Sustainable entrepreneurship duration was explored through the continuation of businesses rates. After considering the involved elements, a theoretical model was proposed that relates environmental and economic factors to sustainable entrepreneurship over time. The study was conducted with data collected from 50 countries around the world from the Global Entrepreneurship Monitor (GEM) 2017 and Sustainable Development Report. A quantitative analysis was done by the Partial Least Squares technique to validate the proposed model [13]. PLS allows construct reliability, convergent validity and discriminant validity tests to be explored in proposed models, and has been used in an increasingly number of organisational research works [7,14].

This research contributes to shed light on some factors that influence the durability of sustainable entrepreneurship, and to increase knowledge about how fulfilling certain SDGs impacts sustainable entrepreneurship over time. The beneficiaries of this research are academics, government organisations, entities and agencies related to the United Nations, and those entrepreneurs who wish to contribute to one of the three sustainable development axes.

The remainder of this paper is structured as follows. Section 1 introduces the theoretical background of this study. Section 2 presents the employed methodology and data. Section 3 offers and discusses the results obtained from applying PLS. Section 4 ends the paper with some concluding remarks.

### 1.1 Theoretical background and hypotheses

This section theoretically delves into the long-term sustainable entrepreneurship concept. Likewise, economic and environmental factors are analysed by emphasising the SDGs related to them. With this theoretical construction of these main addressed elements, the theoretical model to be validated in the following sections was developed.

### 1.2 Sustainable entrepreneurship over time

Sustainable entrepreneurship merges the sustainability concept with entrepreneurial activity. It focuses mainly on preserving nature, life support and community welfare by searching for opportunities in the environment/market to develop products and services with or without economic gains [6]. Sustainability can be analysed through economic factors, social factors and environmental factors [15]. Economic factors involve indicators related to employment, increasing sales, income stability and profitability [15,16]. Social factors involve measures related to basic needs, social recognition, empowerment, freedom, control and child labour. Environmental factors collect elements related to water and energy use, waste and emissions, waste management, space management and hygiene [16]. Environmental, social and economic factors also involve SDGs, formulated to solve the main problems that affect the world’s population [17,18]. The perspective of these goals is to focus on key areas and to work on them as they profoundly affect the human well-being of present-day and future generations [19].

Accordingly, sustainable entrepreneurship refers to all those ventures that imply considerable concern about environmental, social and economic issues. This entrepreneurship stream has values and a vision that look to the future whose operations are performed for a sustainable purpose [20].

One of the characteristics that defines sustainable entrepreneurship is its long-term approach because the decision-making process has a long-term horizon [21]. Sustainable entrepreneurship sustained over time involves society being committed to respect and properly regulate the use of resources by always thinking about long-term well-being [10]. Such sustainable entrepreneurship does away with the bad practices of other entrepreneurships, who have been continuously criticised because they think only about their own enrichment and not society’s welfare and, thus, neglect the sustainable approach [1].

Sustainable entrepreneurship with a long-term focus involves socially responsible behaviour because it recognises that it is not feasible to use resources indiscriminately in the short term. This is why such entrepreneurships seek innovative alternatives to allow both individual and society development [10]. The value systems of these entrepreneurs guide them towards the goal of future sustainable action. Similarly, sustainable entrepreneurship takes a strong ethical and moral basis by recognising commitment to society and future generations as a moral duty [22]. By considering that ethics and social commitment prevail in these entrepreneurships, they seek solutions to the social, economic and environmental problems that actually correspond to current needs and future ones.

This is why sustainable entrepreneurships consider performance over time to be relevant by their companies maintaining an adequate economic, social and environmental balance [10], and by respecting future generations’ needs. Support for economic development by sustainable entrepreneurship is based on enhancing the development of physical capital by reducing exploitation levels, promoting investment plans and increasing the efficiency of services and generated products [6]. Likewise, sustainable entrepreneurs are able to reduce environmental issues by preserving ecosystem integrity.

### 1.3 Environmental factor

In the present research, the environmental factor is recognised as a system that includes the ecosystem. It resembles the definition by [23], where the environmental factor is characterised as an environmental system that enhances the well-being of both individuals and the planet in conjunction with business profits.

Accordingly, the environmental factor includes issues related to water and sanitation, life on land, climate change and clean energy. In this context, SDG6 is related to access clean water and sanitation. Access to drinking water is a global problem because about 1 billion people have no access to it [24]. By considering the need to face water problems, sustainable entrepreneurship over time has developed proposals to solve these issues [25–27]. For example, eco-innovations have been implemented to transform contaminated water into drinking water by using economical technologies [11], and by implementing clean technologies by rationally using resources with an impact, like reducing water and fossil fuel uses [28]. The recovery of exhausted fish populations has been implemented thanks to sustainable entrepreneurship alternatives [11].

The energy problem is another issue of environmental factor content in SDG7 as most of the world’s population has no access to electricity [29]. Thus the planning, organisation, financing and maintenance of renewable energy and energy-saving projects are alternatives in which sustainable entrepreneurship over time can work [30]. Other possible solutions to be implemented by sustainable entrepreneurship are projects that have to do with not only wind, solar, hydro and biomass energies to reduce energy use [30], but also with eco-facilities implementations like efficient buildings or green buildings [31,32].

Climate change is another problem related to SDG13, which has been intensified by human greenhouse gas emissions [33]. Thus a change in climate policies is necessary, in which a shift to a low-carbon economy through technological modifications is possible [34]. In this scenario, sustainable entrepreneurship has the chance to make environmental transformations [33–35], and the development of carbon-free products is an example of another project to be undertaken [30].

Other environmental factor problems caused by inadequate business practices are deforestation and biological degradation, which go against the terrestrial ecosystem [29]. Here SDG15 focuses on problems with life on land. Long-term sustainable entrepreneurship can remove certain activities as solutions to preserve ecosystems by, for example, eliminating environmental degradation [36]. Focusing on eradicating deforestation and boosting agricultural practices can promote biodiversity [36–38]. Sustainable entrepreneurship is able to generate products and services that save ecosystems by developing a mechanism to sustain nature and ecosystems [37].

The environmental factor is related directly to the promotion and durability of sustainable entrepreneurship [39], which takes us to the first hypothesis:

*H1*: *Hypothesis 1*: *There is a positive relation between environmental factors and entrepreneurship sustainable over time*

### 1.4 Economic factors

The economic factor strongly impacts entrepreneurship development, regardless of it being sustainable or not. In flourishing economies, entrepreneurships are strengthened as people are encouraged and wish to become their own bosses, and they take advantage of economic strength to enhance their ventures. Previous studies have confirmed that income levels affect entrepreneurial activity as the rate at which income grows positively affects entrepreneurship rates [40]. This is because economic development can lead to new ventures being created and can strengthen already consolidated ventures as there are more chances of entrepreneurship succeeding [41]. Moreover, when the economic factor deteriorates, it is usually difficult for entrepreneurships to survive and maintain their operations. So they close and their owners become employees paid to subsist [40].

This is why the economic factor has been identified as a key element to enhance sustainable entrepreneurship durability [42]. For example, it has been recognised that access to capital through organised public securities markets is not only an excellent incentive to entrepreneurship, but also a vibrant economy to encourage the durability of these ventures [42]. The political stability and economic politics that support a positive transparent investment climate strongly influence sustainable entrepreneurship outcomes and their durability and stability over time [39].

The economic factor is reflected in some SDGs. For example, SDG1 defines poverty eradication. Poverty is reflected in half the world’s population with people earning less than 2 dollars/day, and almost 1 billion people live on less than 1 dollar/day [43]. As possible actions, sustainable entrepreneurship can develop programmes to provide microcredits and microfinance through microcredit banking [30]. These microcredits help to reduce poverty by providing the poor and people on low incomes, especially women, with small loans at low interest rates [44].

SDG8 focuses on decent work and economic growth. Labour force is an issue due to higher unemployment and child labour rates, and is also due to precarious work with the protection of informal activities predominating [45]. As sustainable entrepreneurship with long-term scopes will be a greater concern in the socio-economic future, it will focus on offering productive employment and decent work [46]. This is possible thanks to a consolidated economy after eliminating demeaning malpractice patterns and abolishing exploitation [45]. Sustainable entrepreneurship not only benefits from economic development, but is considered an economic engine because it boosts decent employment through new ventures that provide people with goods and services, which enhances economic growth [47–49]. Sustainable entrepreneurship with long-term performance can create jobs with decent working conditions and fair wages [30] because one of the premises for eradicating poverty is paying attention to create decent jobs [45].

Another goal related to economy is SDG9, which refers to industry, innovation and infrastructure. Sustainable entrepreneurship over time is able to develop clean sustainable industry innovations and infrastructures, and can generate innovative processes that involve, for example, marketing techniques and managing supply chains [50]. As an initiative, sustainable entrepreneurship has the potential to generate sustainable industry sites and parks, as well water infrastructures [30].

Hence sustainable entrepreneurship durability over time is markedly induced by the existing economic factor’s level. So, the second hypothesis posits that:

*H2*: *Hypothesis 2*: *There is a positive relation between the economic factor and sustainable entrepreneurship over time*

**Fig 1:**
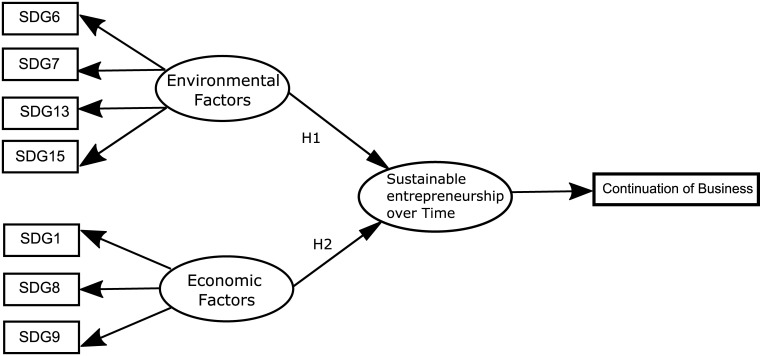
Research model. Source: Authors’ elaboration.

In line with the aforementioned hypothesis, the model herein proposed is shown in Fig 1. This model is proposed to confirm the direct and positive impacts that environmental and economic factors have on sustainable entrepreneurship over time.

## 2. Research methodology

### 2.1 Data collection, sample and measures

For this research, information was collected about consolidating economic and environmental factors in different countries, as were data on the durability of their sustainable entrepreneurship. In this way, the database to be used from different regions was taken into account to represent the existing reality by incorporating countries with distinct levels of economic and social development. This allowed the largest number of geographic areas to be covered so that the obtained results could be globally extrapolated. So this study was conducted with data collected from 50 countries around the world. Data were acquired from the Global Entrepreneurship Monitor (GEM) 2017 [51] and Sustainable Development Report (SDG Index & Dashboards), available online. Both databases contain information about more countries, but only 50 countries presented information in both databases during the match process. The outcome of the data recollection process is shown in Table 1.

**Table 1:** Countries’ data.

Country	SDG1*	SDG6*	SDG7*	SDG8*	SDG9*	SDG13*	SDG15*	Discontinuation of businesses
Argentina	99,8	98.5	84.4	71.7	40.5	85.6	52.4	3.0
Australia	99,7	97.5	84.1	81.8	83.2	30.1	55.0	3.8
Bosnia and Herzegovina	100,0	98.0	65.7	49.4	24.4	86.2	59.6	1.3
Brazil	94,6	93.9	89.7	61.3	46.2	87.2	58.2	5.3
Bulgaria	98,4	86.9	86.5	63.6	37.1	82.4	90.4	1.3
Canada	99,6	88.1	91.2	83.7	75.1	66.0	51.2	6.9
Chile	100,0	98.1	86.9	70.9	43.0	89.7	48.7	7.1
China	99,5	88.2	67.7	71.9	57.7	58.7	58.5	2.8
Colombia	93,7	89.1	83.7	55.0	28.7	87.0	54.0	6.5
Croatia	99,2	95.7	85.5	70.6	46.6	88.8	81.3	4.0
Cyprus	99,9	89.9	85.9	73.8	39.6	68.1	81.6	4.3
Ecuador	94,7	87.7	81.5	65.3	25.3	88.1	57.5	8.8
Egypt	99,6	71.6	89.2	49.8	30.5	88.5	56.9	10.2
Estonia	99,2	96.3	81.4	79.5	61.0	75.1	78.7	4.4
France	99,7	95.4	94.6	79.7	77.2	79.2	73.4	3.3
Germany	99,8	93.3	87.7	85.2	81.0	84.3	77.4	1.6
Greece	97,6	94.7	86.2	66.1	46.2	70.6	73.6	5.1
Guatemala	90,4	82.4	57.0	60.7	13.4	88.7	43.9	6.0
India	93,4	73.7	54.0	68.3	33.1	74.7	47.0	3.2
Indonesia	94,6	81.6	64.8	67.7	25.4	88.5	44.2	4.8
Iran	100,0	68.4	78.2	71.4	26.7	73.4	56.7	6.6
Ireland	99,7	90.2	86.4	82.4	70.5	85.7	74.6	3.3
Israel	99,5	74.8	88.6	82.7	69.7	83.1	31.3	4.8
Italy	98,5	90.9	88.0	79.0	60.7	75.7	78.2	2.1
Japan	99,3	94.3	87.8	92.1	87.3	80.0	64.9	1.5
Kazakhstan	100,0	92.4	74.0	75.0	36.9	75.6	45.7	7.5
Latvia	98,9	92.2	84.6	85.3	42.8	78.8	78.3	4.2
Lebanon	95,5	80.5	87.5	75.6	35.4	77.7	50.7	6.6
Luxembourg	99,6	88.1	66.7	92.5	64.6	72.8	50.9	3.2
Madagascar	0,0	44.4	4.2	42.8	6.3	88.0	55.5	6.7
Malaysia	98,2	90.0	84.1	71.2	60.8	82.4	31.6	8.3
Mexico	97,9	87.5	79.4	70.3	38.5	85.0	42.0	3.5
Morocco	99,6	77.6	76.2	59.3	30.7	84.1	70.3	4.5
Netherlands	99,5	93.5	84.8	87.8	83.0	65.9	74.6	3.1
Panama	98,0	85.1	80.1	71.4	28.8	82.5	53.5	2.7
Peru	97,8	84.6	74.6	52.2	23.3	84.1	58.7	6.2
Poland	100,0	95.2	81.4	86.3	46.7	82.9	83.9	2.8
Qatar	99,8	49.4	78.4	80.6	50.1	58.6	40.1	5.8
Saudi Arabia	99,2	57.6	82.7	74.4	51.7	73.0	35.3	8.8
Slovenia	99,8	94.2	90.6	75.7	56.4	88.3	80.5	2.3
South Africa	66,4	81.8	71.7	37.5	45.1	79.4	44.4	6.0
Spain	98,4	91.5	91.2	80.9	66.9	84.9	58.3	1.9
Sweden	99,4	95.2	97.5	91.3	89.6	80.1	63.1	2.5
Switzerland	99,9	95.1	94.4	85.6	93.9	81.9	66.5	1.1
Thailand	100,0	95.1	76.9	85.2	39.8	73.0	63.2	9.2
United Arab Emirates	99,4	50.2	82.9	84.5	61.8	48.0	29.5	9.2
United Kingdom	99,8	94.3	87.8	84.6	80.7	74.9	64.5	2.6
United States	99,3	96.1	87.4	85.5	84.4	54.2	44.6	4.0
Uruguay	99,9	98.2	94.6	71.3	35.5	78.0	35.5	5.0
Vietnam	99,0	90.7	72.4	60.8	24.9	73.4	46.6	4.2

* Average score on each sustainable development goal

Source: Authors’ compilation based on Global Entrepreneurship Monitor (GEM) 2017 and 2017 SDG Index Report

To describe sustainable entrepreneurship over time, the Discontinuation of Businesses Indicator was taken from the GEM. This measure reflects those “people who have closed, sold or discontinued their business in the last 12 months” [52] and is expressed as a percentage (discontinued entrepreneurship/total entrepreneurship *100). During information processing, it was necessary to obtain the Complementary Percentage of this indicator (100- Discontinuation of businesses indicator) to obtain the missing percent for the Continuation of Businesses rate. Therefore, the data used to validate the proposed model included the Continuation of Businesses indicator to reflect the degree at which entrepreneurships are sustained over time and them becoming sustainable entrepreneurships over time. So the Continuation of Businesses indicator is the variable that measured sustainable entrepreneurship.

To analyse the economic and environmental factors, some SDGs were addressed. In this case, data from SDG Index reports countries’ performance on historic Agenda 2030 and SDGs were used. The SDG Index is pioneering for studying the extent to which each country fulfils SDGs [53]. For the present research work, the SDG data were taken from the 2017 SDG Index Report and collected data were about environmental and economic factors. In the environmental factors analysis, data were collected about SDG6 Clean water and sanitation, SDG7 Affordable and clean energy, SDG13 Climate action and SDG15 Life on land. In the economic factors case, data were compiled about SDG1 No Poverty, SDG8 Decent work and economic growth, and SDG9 Industry, Innovation and Infrastructure. Consequently, the variables SDG6, SDG7, SDG13, SDG15 allowed the construct Environmental factors to be measured. The variables SDG1, SDG8 and SDG9 make it possible to quantify the construct Economic Factors.

Table 2 shows the descriptive statistics.

**Table 2:** Descriptive statistic.

	Mean	Standard Deviations	Minimum	Maximum
Environmental Factors				
SDG6 Clean water and sanitation	86.39	12.96	44.4	98.5
SDG7 Affordable and clean energy	80.45	14.43	4.2	97.5
SDG13 Climate action	77.47	11.70	30.1	89.7
SDG15 Life on land	58.34	15.41	29.5	90.4
Economic Factors				
SDG1 No poverty	95.91	14.71	0.0	100.0
SDG8 Decent work and economic growth	73.02	12.83	37.5	92.5
SDG9 Industry, Innovation and Infrastructure	50.17	22.01	6.3	93.9
Continuation of Business	95.32	2.35	89.8	98.9

Source: Authors’ elaboration

### 2.2 Statistical method: Partial least squares

To validate the model, a structural equation model (SEM) was employed, which is a useful multivariate analysis in Social Science research. The uniqueness of SEM is that it combines a factor analysis and linear regression models to test theories [54]. By this analytical approach, it is possible to analyse relations between latent variables (non-observable variables) that represent theory concepts and data recollect through indicators [54].

The SEM technique herein used was PLS, which takes a covariance-based approach [13]. This technique offers main features for the present study, including not requiring the variable metric uniformity, and it can estimate models with small samples [55].

Several studies about sustainable entrepreneurship have applied PLS because PLS is a feasible method for exploring construct reliability, convergent validity and discriminant validity tests in proposed models [7]. This technique has been used in an increasingly number of organisational research works about sustainability and entrepreneurship. Studies like [7,14,56,57] incorporate the PLS method to explore causal relations in the explored context, sustainable entrepreneurship where, for example, [57] analysed the effect of social, cultural and economic factors on entrepreneurship. SmartPLS 3 was the software employed for latent variable modelling [58].

In the research we followed the steps suggested by [59] and [60]:

1. Construction of the nomological network, where constructs are included (economic factor, environmental factor and sustainable entrepreneurship over time) along with the variables involved (SDG1, SDG6, SDG7, SDG8, SDG9, SDG13, SDG15). Through the nomogram, it is possible to explicitly specify both the structural model (internal model) and the relations between the indicators and constructs in the measurement model (external model) using a representation of the relations between variables.
2. Assessment of the overall model, where the model fit test is performed and it is possible to prove if data are coherent with a factor model. This analysis is reinforced in Subsection 2.3.
3. Assessment of the validity and reliability of the measurement model. The measurement model attempts to analyse if the theoretical concepts are measured correctly through the observed variables. This analysis is reinforced in Subsection 2.4.
4. Assessment of the structural model. The structural model evaluates the weight and magnitude of the relations between different variables. This analysis is reinforced in Subsection 2.5.

### 2.3 Assessment of the global model fit

One of the steps in the PLS is to analyse the global model fit. The global model fit assessment was done to analyse if the estimated model fitted the data by dismissing the possibility of data containing more information than those the model provided [60]. The model fit test involves fit indices and inferential statistics through bootstrap-based tests for estimated models [60]. The applied model fit criterion is the standardised root mean square residual (SRMR), where a value under 0.08 is considered an acceptable fit [61]. The bootstrap-based tests of an overall model fit include the bootstrap-based inference statistics of SRMR (SRMR ≤ HI95 ≤ HI99), unweighted least squares discrepancy (d_ULS_ ≤ HI95 ≤ HI99) and geodesic discrepancy (d_G_ < HI95 < HI99) [60], where the non-compliance of one of these measures implies that the model does not adequately fit [62].

### 2.4 Assessment of the measurement model

Another analysis in the PLS involves assessing the measurement model. The measurement model analysis provides empirical measures of the relations between indicators (SDG1, SDG6, SDG7, SDG8, SDG9, SGD13 and SDG15) and constructs (economic factors, environmental factors and sustainable entrepreneurship over time). The confirmatory factor analysis (CFA) of the saturated model is analysed with fit indices (SRMR ≤ 0.08) and inferential statistics through bootstrap-based tests for saturated models (SRMR ≤ HI95 ≤ HI99; d_ULS_ ≤ HI95 ≤ HI99; d_G_ < HI95 < HI99) [60].

In our study, the proposed model had reflective indicators (Mode A) when considering that all the indicators “can be viewed as a representative sample of all the possible items available within the conceptual domain of the construct” [63]. The most important metrics applied to assess the measurement model in reflective modes are reliability, convergent validity and discriminant validity [64]. The reliability of each measure is assessed through the indicators loading (λ), which must be above 0.707 (λ ≥ 0.707). Construct reliability (internal consistency) is assessed with Cronbach’s alpha (≥ 0.7) [64], composite reliability (ρc ≥ 0.7) [65] and Dijkstra-Henseler’s indicator (ρ_A_ ≥ 0.7) [66].

Convergent validity is assessed by the average variance extracted (AVE ≥ 0.5) [65]. Discriminant validity is analysed using cross loadings, where no item should load more heavily on another construct than on that construct that it attempts to measure [67]. Other convergent validity indicators are Fornell-Larcker criteria (AVE square root > correlation among the other constructs) [68] and the Heterotrait-monotrait ratio (HTMT ≤ 0.85 ≤ 0.9) [69].

### 2.5 Assessment of the structural model

After confirming that the measurements of constructs are reliable, the assessment of the structural model is made to assess the model’s predictive capacity. The principal analyses done to assess the structural model are collinearity, path coefficient sign and magnitude, significance of path coefficient, determination coefficients and effect size. Collinearity is tested through the variance inflation factor (VIF <5) [65]. Standardised paths should take values over 0.20 to be considered relevant [65], and a determination coefficient (R^2^) is acceptable when it exceeds 0.25 for each construct [70]. Values for effect size (f^2^) between 0.02 and 0.15 are considered weak, those between 0.15–0.35 are moderate and values above 0.35 indicate strong effects [64].

## 3. Results

After assessing the global model fit, the principal results obtained were that SRMR was 0.041 and took a value under 0.08. SRMR was also lower than the 95% bootstrap (0.081) and the 99% bootstrap (0.104) quantiles. Moreover, the d_ULS_ (0.025) value went below the 95% bootstrap (0.098) and the 99% bootstrap (0.162) quantiles, and that of d_G_ (0.010) was under the 95% (0.049) and the 99% (0.073) bootstrap quantiles. Hence the model fitted the data.

With the CFA of the saturated model, the measurement model assessment showed that all the indicators had acceptable values; i.e., SRMR = 0.041≤ 0.08, SRMR ≤ 0.081 (HI95) ≤ 0.096 (HI99); d_ULS_ = 0.025 ≤ 0.098 (HI95) ≤ 0.137 (HI99); d_G_ = 0.010 < 0.047 (HI95) < 0.068 (HI99). These values confirmed the aforementioned idea of the model well fitting the data.

The reliability analyses detected that the loading of three indicators was under 0.707, while SDG1, SDG7 and SDG13 had λ values of about 0.543, 0.531 and 0.212, respectively (Table 3). These indicators were removed from the model to not compromise the reliability requirement. So, the reliability analysis was carried out again. This time all the factor loadings obtained an acceptable value above 0.707 (λ_SDG6_ = 0.833, λ_SDG8_ = 0.916, λ_SDG9_ = 0.952, λ_SDG15_ = 0.857). The constructs reliability research detected that the Environmental Factors construct had a Cronbach’s alpha of 0.600, below 0.7. Fortunately, the other two constructs (Economic Factors and Sustainable Entrepreneurship over time) obtained adequate values, with 0.857 and 1, respectively. When examining the reliability analysis in-depth, composite reliability indicators ρc obtained good values over 0.7 for the three constructs (ρ_c_ Environmental Factors = 0.833; ρ_c_ Economic Factors = 0.932; ρ_c_ Sustainable Entrepreneurship over time = 1). The last criterion addressed in the reliability analyses was Dijkstra-Henseler’s indicator ρ_A_, with good values over 0.7 for two constructs: ρ_A_ Economic Factors = 0.903; ρ_A_ Sustainable Entrepreneurship over time = 1. The remaining Environmental Factor had a lower value (ρ_A_ = 0.602). As composite reliability is more appropriate than Cronbach’s alpha for PLS because it does not assume that all the indicators receive the same weighting [71], the lower Cronbach’s alpha value of the Environmental Factors did not interfere with the confirmed reliability. Thus constructs reliability was confirmed, which implied the consistency of the measures employed in the proposed model.

**Table 3:** Composites and measures.

	Outer loadings 1st Model	Outer loadings 2nd Model*	Cronbach´s alpha	ρ_A_	ρ_c_	AVE
Enviromental Factors			0.600	0.602	0.833	0.714
SDG6 Clean water and sanitation	0.849	0.833				
SDG7 Affordable and clean energy	0.531	-				
SDG13 Climate action	0.212	-				
SDG15 Life on land	0.823	0.857				
Economic Factors			0.857	0.903	0.932	0.873
SDG1 No poverty	0.543					
SDG8 Decent work and economic growth	0.936	0.916				
SDG9 Industry, Innovation and Infrastructure	0.907	0.952				

*The 2nd model did not include SDG1, SDG7 and SDG13

Source: Authors’ elaboration

Convergent validity was confirmed by the AVEs over 0.5 for Environmental Factors, Economic Factors and Sustainable Entrepreneurship over time (0.714, 0.873 and 1, respectively). This indicates that each set of indicators represents a single construct because the variance explained by the variables was higher than the variance accounted for by the measurement error. Discriminant validity was verified through cross loadings, where each item load was heavier towards the construct that it attempted to measure (see Table 4). Likewise, convergent validity was confirmed with Fornell-Larcker criteria and Heterotrait-monotrait ratio compliance. For example, the AVE square root of each construct was higher than the correlation between the other constructs ($\sqrt[{2}]{{AVE}_{green\;factor}}=0.845>0.288\;\text{and}\;0.288$ and 0.581; $\sqrt[{2}]{{AVE}_{economic\;factor}}=0.934>0.385\;\text{and}\;0.288$) (see Table 5). With HTMT, all the values were under 0.9 (HTMT_Environmental Factors-Economic factors_ = 0.402; HTMT_Environmental Factors-Sustainable entrepreneurship over time_ = 0.749; HTMT_Economic Factors-Sustainable entrepreneurship over time_ = 0.408). The confirmation of discriminant validity reflected that the three constructs differed from one another, which thus verified the reliable constructs measurements.

**Table 4:** Discriminant validity. Cross loadings analysis.

	Environmental Factors	Economic Factors	Sustainable Entrepreneurship over time
SDG6 Clean water and sanitation	0.833	0.319	0.473
SDG15 Life on land	0.857	0.173	0.508
SDG8 Decent work and economic growth	0.245	0.916	0.306
SDG9 Industry, Innovation and Infrastructure	0.289	0.952	0.402
Continuation of Business	0.581	0.385	1

Source: Authors’ elaboration

**Table 5:** Discriminant validity. Fornell-Larcker criteria.

	Environmental Factors	Economic Factors	Sustainable Entrepreneurship over time
Environmental Factors	0.845		
Economic Factors	0.288	0.934	
Sustainable Entrepreneurship over time	0.581	0.385	1.00

* Diagonal values represent the AVE square root of each construct. The remaining values are correlations.

Source: Authors’ elaboration

**Fig 2:**
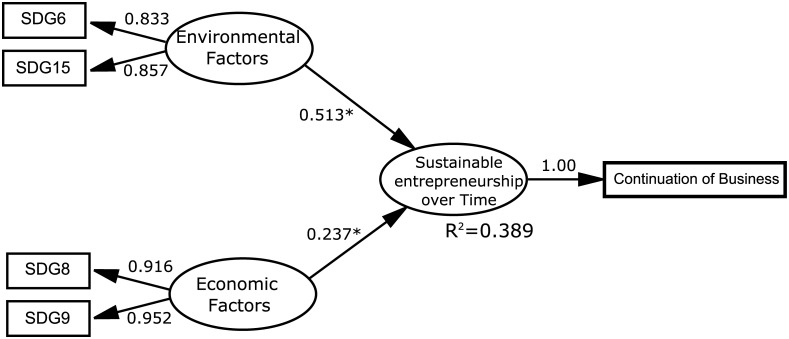
Results for the model. Source: Authors’ elaboration.

The structural model assessment exhibited the non-existence of multicollinearity in either of the two relations because VIF was below 5 (VIF_Environmental Factors_ = 1.09; VIF_Economic Factors_ = 1.09). Likewise, all the standardised paths had values over 0.20 (see Fig 2), and were recognised as relevant. The higher standardised path was that which related environmental factors with sustainable entrepreneurship over time. This implied that environmental factors more strongly influenced sustainable entrepreneurship over time than economic factors. The two path coefficients were significant (p_value_ <0.05) and positive, which agrees with the theoretical postulates. This was also shown by the 5% and 95% confidence intervals, where relations did not reach zero and, thus, denotes low variability. The R_squared_ was 0.389, which is a moderate value and implied that Environmental and Economic factors explained about 40% of the performance of sustained entrepreneurship over time. The effect size analysis showed that Economic factors had a weak effect on Sustainable Entrepreneurship over time (f^2^ = 0.084), and that the Environmental factors effect was strong (f^2^ = 0.394), which implied that Environmental factors explained more Sustainable entrepreneurship over time in R_squared_ terms. Therefore, the predictive capacity of the proposed model was good because its measures were reliable and valid, and the relations between constructs were close and significant.

## 4. Discussion

The objective of this work was to create a model in which the environmental and economic factors included in SDGs are related to sustainable entrepreneurship over time, specifically to a business created by an entrepreneur lasting.

To confirm the possible direct impacts and their sign on these two groups of sustainable entrepreneurship factors, a structural equations model (SEM) was build based on PLS by a covariance-based approach. This study was conducted using data collected from 50 countries in diverse geographic areas. These data were obtained from the GEM and the Sustainable Development Report. To describe sustainable entrepreneurship over time, an indicator of discontinued business in the GEM was taken and the complementary percentage of this indicator was calculated.

Analysing the model allowed us to determine (validate) the considered relations by a theoretical framework study with the following indicators: SDG1, SDG6, SDG7, SDG8, SDG9, SGD13 and SDG15, and with three constructs: environmental factors, economic factors and sustainable entrepreneurship over time.

The proposed model has good predictive capacity, its measures are reliable and valid, and the relation between constructs is sound and significant. Each set of indicators represented a single construct and all three constructs differed from one another, which implies that they are reliable.

In line with the hypotheses herein set out, the results revealed a significant positive relation among the factors obtained from the environmental and economic SDG with sustainable entrepreneurship over time, and both factors explained almost 40% of sustainable entrepreneurship performance. Moreover, the environmental factors more strongly influenced sustainable entrepreneurship with time, whose effect can be considered substantial.

Therefore, Hypothesis 1 was confirmed because a significant and positive relation between environmental factors and sustainable entrepreneurship over times was validated, which falls in line with that stated in [39] and [12].

According to the performed analyses, which environmental SGD met the criteria to be considered in the model were detected. This indicated that protect, restore and promote the sustainable use of terrestrial ecosystems (SDG15) would remain in the model, which falls in line with the works of [36], and links this entrepreneurship with not only performing more activities that stop environmental degradation, but also with better agricultural practices to promote biodiversity [37].

Availability of water and sanitation (SDG6) would also remain, with a relation to sustainable entrepreneurship, as pointed out by [11,25,27], insofar as creating business opportunities in sectors like implementing technology for providing drinking water and reducing water use.

The variables related to accessing available safe energy (SDG7) and taking urgent measures to fight climate change (SDG13) did not fulfil the criteria to be left in the model. This means that no significant effect of both factors on sustainable entrepreneurship over time was detected in this model.

The positive and significant effect of economic factors on sustainable entrepreneurship with time was verified, which falls in line with what Hypothesis 2 states. This result agrees with the works by [39,42], which point out the importance of the economic factor for lasting entrepreneurship over time.

For economic factors, SDG8 met the conditions to remain in the model; that is, decent work and economic growth were related to sustainable entrepreneurship, mainly by creating new companies that increase job opportunities with fair salaries and, thus, in turn helps economic growth. This coincides with the works of [45–49]. The result herein obtained also coincides with the work of [30], which evidenced a relation between sustainable entrepreneurship over time and creating jobs/decent salaries. Consequently, lasting sustainable entrepreneurship over time is due to the level of the existing economic factor to a great extent.

The objectives of building infrastructures, promoting sustainable industrialisation and favouring innovation (SDG9) also remained in the model. This relation was also found by [30] for water-related infrastructures, and also by [50] for aspects of marketing techniques and managing supply chains. Generally speaking, political stability and adopting economic policies that support a positive investment background with a future influence sustainable entrepreneurship and it lasting, which has also been pointed out by [39]. The goal to eradicate poverty (SDG1) did not, however, meet the requirements to remain in the model.

## 5. Conclusions

Therefore, one general outcome of our research is that those countries which invest in, and make more efforts, to consolidate their environmental and economic factors tend to show long-lasting rates for their sustainable entrepreneurship over time. The interest of this work is not only for the academic field given its contribution to the SDG research current or to sustainable entrepreneurship, but also for governmental organisations, entities and agencies related to United Nations, and for those entrepreneurs who seek a business opportunity in relation to SDG.

We believe that the present research work provides governments and agencies that work to meet the SDGs with information to help them to guide their cooperation actions and investments by credits, subsidies and aid in those SDG that are positive and significant. Thus by their actions, they could empower long-lasting sustainable entrepreneurship and, with it, quality jobs, economic growth and social well-being by following our proposal of identifying those SDG that are strategic for this purpose.

In this way, governments and public administrations have to combine efforts to fulfil the SDG related to the natural environment and the economy. This would ensure better infrastructures and industry, and decent jobs, and entrepreneurship would persist with time. Moreover, if these institutions invest in supplying cleaner water and improving life in the terrestrial ecosystem, it would have a positive effect on long-term entrepreneurship.

Researchers could also benefit because the support and financing lines that can be set up in accordance with this strategy require continuous research. Our research could be the first step to not only identify SDG, but to also measure their outcomes in the creation and duration of companies linked with them by proposing an interesting topical research line. It would also be positive for those entrepreneurs seeking guidance for their business in SDG because this would allow them to know which sectors linked with environmental and economic SDG are related more to companies’ survival.

This study was conducted with the data collected from 50 countries worldwide with different levels of economic, environmental and social development. It is possible to generalise the conclusions because the regions involved in this research are varied, where different geographical areas around the world were covered.

As possible limitations and future lines, we point out that the obtained R^2^ coefficient was acceptable, but could increase when considering further SDG aspects or macroeconomic-type variables. We also believe that it would be interesting to complete our research by studying more years and checking the model’s robustness over time. Likewise, it would be interesting to investigate possible differences in geographical areas, and to include the social aspect of SDGs in a future model.
